# Self-Stigma, Social Stigma, and Attitudes Toward Seeking Psychological Help Among University Students: A Cross-Sectional Study

**DOI:** 10.3390/bs16091597

**Published:** 2026-09-08

**Authors:** Muhammet Sancaktar, Bahadır Demir, Gülçin Elboğa, Feridun Bülbül, Abdurrahman Altındağ

**Affiliations:** Department of Psychiatry, Faculty of Medicine, Gaziantep University, Gaziantep 27310, Türkiye; bdemir@gantep.edu.tr (B.D.); gelboga@gantep.edu.tr (G.E.); feridunbulbul@gantep.edu.tr (F.B.); aaltindag@gantep.edu.tr (A.A.)

**Keywords:** psychological help-seeking, self-stigma, public stigma, university students, attitudes

## Abstract

Stigma is a major barrier to psychological help-seeking among university students. This cross-sectional study examined how self-stigma and social (public) stigma are associated with attitudes toward seeking psychological help, and which sociodemographic characteristics relate to these variables, among students at a large public university in Gaziantep, Türkiye. A total of 457 undergraduates (286 women, 62.6%; mean age 20.6 years) completed the Attitudes Toward Seeking Professional Psychological Help Scale–Short Form (ATSPPH-SF), the Self-Stigma of Seeking Help Scale (SSOSH), and the Social Stigma for Receiving Psychological Help Scale (SSRPH), together with a demographic form. Data were analyzed with *t*-tests, one-way ANOVA, Pearson correlations, and two-step hierarchical multiple linear regression. Self-stigma (r = −0.23) and social stigma (r = −0.15) were both negatively associated with help-seeking attitudes, and the two stigma constructs were positively correlated (r = 0.38). In hierarchical regression, demographic variables explained 9.7% of the variance in attitudes; adding the stigma variables produced a significant increment (ΔR^2^ = 0.039, *p* < 0.001; full model R^2^ = 0.136). Only self-stigma (β = −0.17) and female gender (β = 0.17) remained independent predictors. Women and students with a family history of psychiatric disorder or a previous diagnosis reported more positive attitudes; men reported higher social stigma; and older age was associated with lower social stigma. Faculty and income were not significantly associated with attitudes. Self-stigma appears to be the more proximal stigma-related correlate of help-seeking attitudes, consistent with the self-stigma model of help-seeking. Contact-based and cognitive stigma-reduction efforts targeting self-stigma, together with male-focused outreach, may be useful in university settings.

## 1. Introduction

University enrollment coincides with the developmental transition to emerging adulthood, a period marked by identity consolidation, academic pressure, and increased vulnerability to mental health problems (Kitzrow, 2003). International evidence indicates that psychological distress is common among students yet that only a minority seek professional support; in a large multinational survey, most students experiencing distress did not access care (Eisenberg et al., 2007; Broglia et al., 2021). This treatment gap has persisted, and in some settings widened, in the post-pandemic period (Di Consiglio et al., 2021; Chang et al., 2024).

A consistent explanation for the gap is stigma. Following Corrigan and Watson (2002), it is useful to distinguish public (social) stigma—the negative stereotypes, prejudice, and discrimination that a community directs toward people who seek help or who have mental health problems—from self-stigma, the internalization of these public attitudes, which erodes self-esteem and self-efficacy. Public stigma is not limited to mental illness; comparable processes operate for a range of stigmatized conditions and identities and, in collectivist contexts, extend to the family as an interdependent (affiliate) form of stigma (Clement et al., 2015; Corrigan et al., 2014). The present study is guided by this two-component stigma model, integrated with the self-stigma model of help-seeking proposed by Vogel et al. (2007), in which public stigma is associated with help-seeking attitudes indirectly, through self-stigma. Within a Theory-of-Planned-Behavior framing, attitudes are in turn proximal determinants of help-seeking intentions and behavior. This model yields two testable expectations for cross-sectional data: that self- and public stigma should be positively associated with one another, and that both—particularly self-stigma—should be negatively associated with help-seeking attitudes.

Prior empirical work is broadly consistent with these expectations but is heterogeneous in context and design. In a U.S. undergraduate sample, self-stigma more strongly predicted attitudes than public stigma (Vogel et al., 2007); a meta-analysis of primarily Western samples confirmed that both public and self-stigma relate negatively to help-seeking attitudes and intentions, with self-stigma the more proximal correlate (Yu et al., 2023). Cross-cultural and national studies show that the strength of these associations varies with cultural norms (Nam et al., 2010; Clement et al., 2015). Turkish studies have reported generally cautious help-seeking attitudes and gender differences favoring women (Çebi & Demir, 2020; Topkaya, 2014, 2021), but they have typically focused on single constructs and single faculties. Two gaps therefore remain. First, few studies in the Turkish higher-education context examine self- and public stigma together in a demographically diverse, multi-faculty sample. Second, the sociodemographic correlates of these constructs—gender, age, socioeconomic position, parental education, and personal or family psychiatric history—have rarely been modeled jointly. We included these variables because each has a theoretical or empirical rationale: gender and age shape both stigma and help-seeking (Mackenzie et al., 2019; Nam et al., 2010); socioeconomic position and parental education index differential exposure to mental-health information and services (Li et al., 2020; Potts & Henderson, 2020); and personal or family experience of psychiatric problems increases familiarity, which is generally associated with lower stigma (Corrigan et al., 2014; Perich & Andriessen, 2025).

We also examined faculty of enrollment, because disciplinary environments differ in their exposure to mental-health content and in prevailing peer norms, and prior work has suggested that students in health-related programmes may hold distinct attitudes; testing this formally allows the assumption to be evaluated rather than presumed. Accordingly, the aim of this study was to examine, in a diverse sample of Turkish university students, (a) the associations among help-seeking attitudes, self-stigma, and social stigma, and (b) the sociodemographic characteristics related to these variables. Given the sociocultural context—a collectivist setting in which family attitudes toward mental health are influential and higher education is highly valued—the findings are intended to inform university counselling services and locally tailored stigma-reduction efforts.

## 2. Materials and Methods

### 2.1. Design and Participants

This cross-sectional study was conducted with undergraduate students at a large public university in Gaziantep, Türkiye. Eligibility criteria were: (i) being 18 years of age or older, (ii) being enrolled in an undergraduate program, and (iii) providing informed consent. Recruitment used convenience sampling through campus email lists and course announcements. Of 496 students invited, 490 responded (response rate 98.8%); after screening for eligibility and data quality, 33 respondents were excluded (missing core demographic data, ≥20% missing scale items, or invariant/patterned responding), yielding a final sample of *N* = 457 (286 women, 62.6%; 171 men, 37.4%; mean age 20.6 years, SD 2.3, range 16–37). Faculty distribution is reported in Table 1.

### 2.2. Procedure and Ethics

Data were collected entirely through an online survey form; the questionnaire took approximately 10–15 min to complete. The study was approved by the Non-Interventional Clinical Research Ethics Committee of Gaziantep University (Decision No: 2023/163; date of approval: 10 May 2023) and conducted in accordance with the Declaration of Helsinki. Before participation, students read an information page describing the study’s purpose, the voluntary and anonymous nature of participation, and their right to withdraw at any time without consequence; they then provided informed consent. No directly identifying information was collected, and data were stored securely and accessible only to the research team. To reduce the risk of coercion, participation was solicited through general campus channels rather than during the researchers’ own classes, and no instructor was present during completion.

### 2.3. Measures

**Demographic form.** Age, gender, faculty, monthly household income, parental years of education, family history of psychiatric disorder, and previous personal psychiatric diagnosis were recorded.

**Attitudes Toward Seeking Professional Psychological Help Scale–Short Form (ATSPPH-SF).** Developed by Türküm (2001), this 18-item, 5-point Likert scale assesses attitudes toward seeking professional psychological help; total scores range from 18 to 90, with higher scores indicating more positive attitudes. The scale has demonstrated satisfactory validity and reliability in Turkish samples, and the previously validated Turkish version was used here. Internal consistency in the present study was α = 0.82.

**Self-Stigma of Seeking Help Scale (SSOSH).** Developed by Vogel et al. (2006), this 10-item, 5-point Likert scale assesses the self-stigma associated with seeking psychological help; total scores range from 10 to 50, with higher scores indicating greater self-stigma. The scale has established reliability and construct validity, including in Turkish adaptation studies, and the validated Turkish version was used. Internal consistency in the present study was α = 0.62; this relatively low value is noted as a limitation.

**Social Stigma for Receiving Psychological Help Scale (SSRPH).** Based on Komiya et al. (2000) and adapted for use in Turkish, this 5-item scale assesses perceived public stigma toward receiving psychological help. In the present administration, a 5-point response format was used (total range 5–25), with higher scores indicating greater perceived social stigma. Internal consistency in the present study was α = 0.84.

### 2.4. Statistical Analysis

Analyses were conducted in Python version 3.12.10 (Python Software Foundation, Wilmington, DE, USA) using the pandas (version 3.0.3), NumPy (version 2.4.6), SciPy (version 1.17.1), and statsmodels (version 0.14.6) packages; the figure was produced with Matplotlib (version 3.10.9). Descriptive statistics summarized the sample. Independent-samples t-tests compared scale scores across two-level variables (gender, family history of psychiatric disorder, previous psychiatric diagnosis), and one-way ANOVA with Tukey HSD post hoc tests compared scores across faculty and income categories. Pearson correlations examined associations among the three scales, age, and parental education. Statistical significance was set at *p* < 0.05.

To test the incremental contribution of stigma beyond sociodemographic characteristics, a two-step hierarchical multiple linear regression was estimated with help-seeking attitudes (ATSPPH-SF) as the dependent variable. Step 1 entered the demographic variables (age, gender, mother’s and father’s education, income level, family history of psychiatric disorder, previous psychiatric diagnosis, and faculty, dummy-coded with Medicine as the reference category). Step 2 added the two stigma variables (self-stigma and social stigma) to evaluate their incremental contribution. For each step, we report unstandardized coefficients (B), standard errors, standardized coefficients (β), t-values, exact *p*-values, and 95% confidence intervals, together with R^2^, adjusted R^2^, the omnibus F-test, and the change statistics (ΔR^2^ and F-change). Because attitudes were the sole outcome of theoretical interest, no model treating the stigma scales as dependent variables was estimated.

Sensitivity and power analyses were computed in SciPy (version 1.17.1) using the non-central F and z distributions, following the effect-size conventions of G*Power; no separate power-analysis software was used. For the omnibus test of the full regression model (16 predictors, *N* = 455, α = 0.05), the study had greater than 99% power to detect the observed effect (R^2^ = 0.136; f^2^ = 0.157) and 80% power to detect an effect as small as f^2^ = 0.044 (equivalent to R^2^ ≈ 0.042). For the incremental test of the two stigma variables (u = 2), power to detect the observed increment (ΔR^2^ = 0.039; f^2^ = 0.045) was 99%, and the minimum detectable increment at 80% power was ΔR^2^ ≈ 0.021. For the group comparisons, the available sample (286 women, 171 men) provided 80% power to detect a standardized mean difference of d = 0.27, and for bivariate correlations (*N* = 457) the minimum detectable effect at 80% power was r = 0.13. The sample was therefore adequately powered to detect small effects for all planned analyses; detecting a very small incremental effect of f^2^ = 0.02 in the full model would have required approximately *N* = 977.

## 3. Results

### 3.1. Descriptive Statistics and Reliability

The sociodemographic characteristics of the sample are presented in Table 1. Mean scale scores were 60.5 (SD 10.8) for the ATSPPH-SF, 27.4 (SD 5.9) for self-stigma, and 10.3 (SD 3.8) for social stigma. Internal consistency was acceptable for the ATSPPH-SF (α = 0.82) and the SSRPH (α = 0.84) and low for the SSOSH (α = 0.62).

### 3.2. Group Comparisons

Group comparisons are summarized in Table 2. Women reported significantly more positive help-seeking attitudes than men (M = 62.1 vs. 57.9; t = 3.92, *p* < 0.001), whereas men reported higher social stigma (M = 10.8 vs. 9.9; t = −2.54, *p* = 0.011); the sexes did not differ on self-stigma. Students with a family history of psychiatric disorder reported more positive attitudes than those without (M = 64.1 vs. 59.9; t = −2.81, *p* = 0.006), as did students with a previous psychiatric diagnosis (M = 63.5 vs. 60.2; t = −2.03, *p* = 0.047); these two groups did not differ significantly on the stigma scales.

Across faculties, attitudes did not differ significantly (F(7, 449) = 1.96, *p* = 0.059), nor did self-stigma (*p* = 0.14). Social stigma did differ by faculty (F(7, 449) = 3.30, *p* = 0.002); it was lowest among medical students (M = 8.8), who differed significantly from engineering students in post hoc comparison (Tukey *p* < 0.01). Income group was not significantly associated with attitudes (F(2, 454) = 0.93, *p* = 0.39) or self-stigma (*p* = 0.15), with only a borderline association with social stigma (F(2, 454) = 3.01, *p* = 0.050).

### 3.3. Correlations

**Figure 1:**
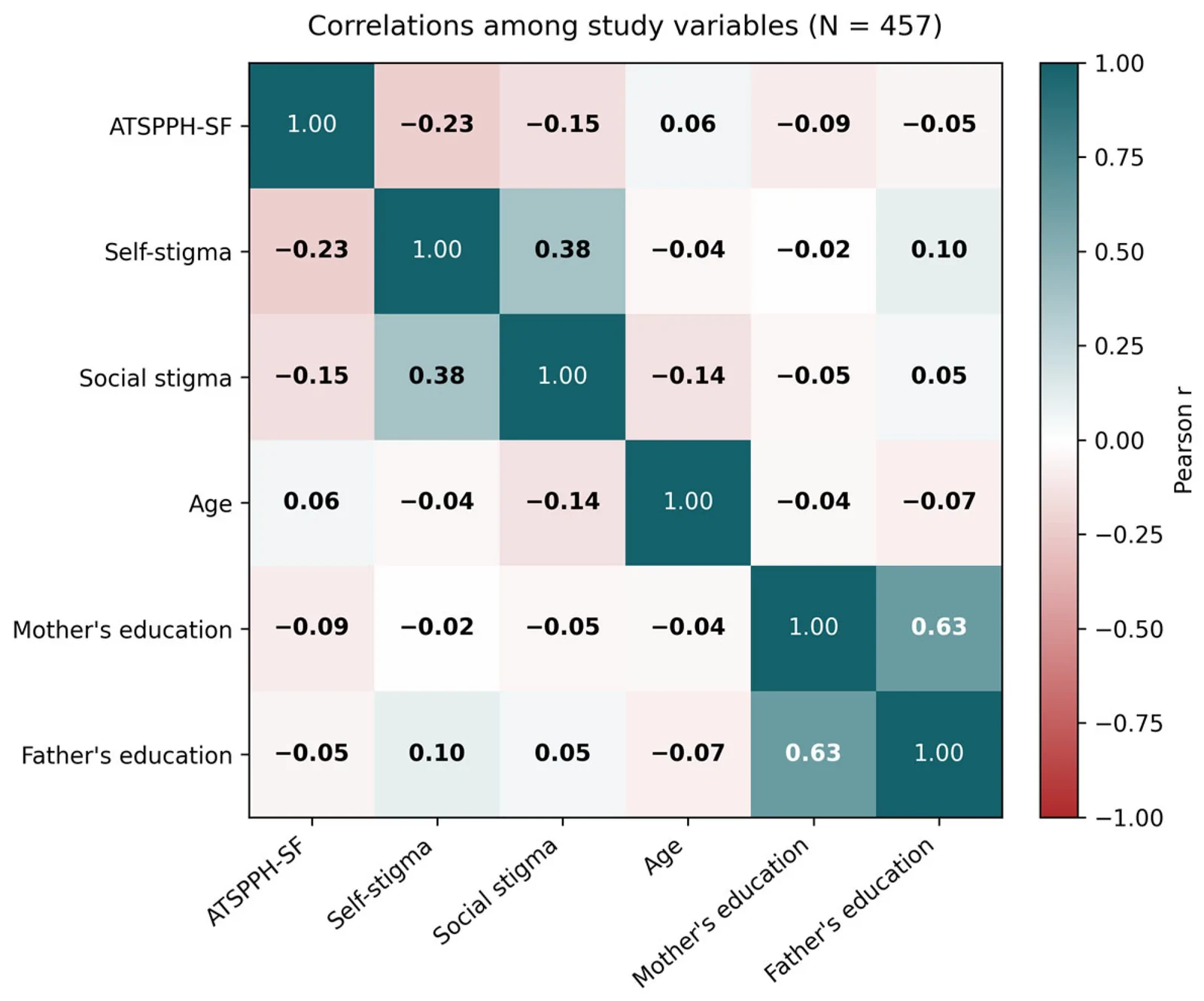
Pearson correlations among the study variables (*N* = 457).

Correlations among the study variables are shown in Figure 1. Help-seeking attitudes were negatively correlated with both self-stigma (r = −0.23, *p* < 0.001) and social stigma (r = −0.15, *p* = 0.001). The two stigma constructs were positively correlated with each other (r = 0.38, *p* < 0.001). Older age was modestly associated with lower social stigma (r = −0.14, *p* = 0.002), whereas attitudes were not significantly associated with age. Parental education showed only weak associations with the study variables.

### 3.4. Regression Analyses

The hierarchical regression results are presented in Table 3 (*n* = 455 with complete data). In Step 1, the demographic block—including faculty—explained a significant but modest proportion of the variance in help-seeking attitudes, R^2^ = 0.097, adjusted R^2^ = 0.068, F(14, 440) = 3.38, *p* < 0.001. Within this step, female gender was the only significant predictor (B = 4.03, β = 0.18, *p* < 0.001); no faculty contrast differed significantly from the Medicine reference category (all *p* > 0.09).

In Step 2, adding self-stigma and social stigma produced a small but statistically significant increment in explained variance, ΔR^2^ = 0.039, F-change(2, 438) = 9.91, *p* < 0.001, with the full model accounting for R^2^ = 0.136 (adjusted R^2^ = 0.105), F(16, 438) = 4.31, *p* < 0.001. In the final model, only self-stigma (B = −0.31, β = −0.17, *p* < 0.001) and female gender (B = 3.84, β = 0.17, *p* < 0.001) remained significant; social stigma was not an independent predictor once self-stigma was included (B = −0.18, β = −0.06, *p* = 0.205), and no faculty contrast reached significance.

The omnibus F-tests indicate that both models explain significantly more variance in attitudes than would be expected by chance. Nevertheless, the total variance explained is modest: the full model accounts for approximately 14% of the variance in help-seeking attitudes, meaning that roughly 86% remains attributable to factors not measured here—for example mental-health literacy, symptom severity, previous service experience, perceived accessibility and cost of services, and social-network influences. The practical implication is that stigma is a meaningful but partial determinant of attitudes: stigma-reduction efforts are warranted, yet they are unlikely to be sufficient on their own and should be combined with strategies that improve knowledge of, access to, and trust in services.

## 4. Discussion

In a diverse sample of Turkish university students, self-stigma and social stigma were both negatively associated with attitudes toward seeking psychological help, and the two stigma constructs were positively related to one another. In hierarchical regression, stigma explained a small but significant increment in variance beyond demographic characteristics (ΔR^2^ = 0.039), and only self-stigma remained an independent correlate of attitudes once both stigma variables were entered. It should be emphasized that, because the design is cross-sectional, these findings describe associations rather than causal or reciprocal effects.

This pattern speaks directly to the two frameworks that guided the study. First, it is consistent with the self-stigma model of help-seeking (Vogel et al., 2007), which proposes that public stigma influences attitudes indirectly, by being internalized as self-stigma. The observation that social stigma correlated significantly with attitudes at the bivariate level (r = −0.15) but lost significance once self-stigma was entered—while remaining strongly associated with self-stigma itself (r = 0.38)—is precisely the pattern expected if self-stigma carries the influence of public stigma onto attitudes. Our cross-sectional data cannot establish mediation, but they are compatible with it and support the model’s central proposition that self-stigma is the more proximal correlate. Second, within the Theory of Planned Behavior, attitudes are a principal determinant of behavioral intention. Situating our findings in that framework implies that stigma is positioned upstream of intention: by shaping attitudes, self-stigma may indirectly constrain students’ intention to seek help. This also clarifies a boundary of the present study—we measured attitudes rather than intentions or behavior, so the downstream links in the model remain to be tested. Together, the two frameworks suggest a chain in which public stigma is internalized, internalized stigma shapes attitudes, and attitudes in turn inform intention; our results provide cross-sectional support for the middle link of this chain.

Consistent with previous Turkish and international research, women reported more positive attitudes than men, and men reported higher social stigma (Nam et al., 2010; Çebi & Demir, 2020; Topkaya, 2021). The more positive attitudes among students with a personal or family history of psychiatric problems align with the familiarity hypothesis, whereby contact with mental illness is associated with lower stigma and greater openness to help (Corrigan et al., 2014; Perich & Andriessen, 2025). The lower social stigma observed among older students is compatible with maturational gains in mental-health literacy (Mackenzie et al., 2019), although this age-related pattern is a between-person association in cross-sectional data and does not demonstrate change within individuals over time.

Faculty was included in the regression model but no faculty contrast was significant, and the earlier discipline-based claims are therefore not supported. The one faculty-related finding that did reach significance in group comparisons—lower perceived social stigma among medical students—may reflect greater exposure to mental-health content in medical training, but given the number of comparisons it should be interpreted cautiously and replicated. Income was likewise unrelated to attitudes in both the group comparisons and the regression models.

The findings have practical rather than strong theoretical implications, and they point to several concrete directions for university mental health services.

First, because self-stigma rather than perceived public stigma was the independent correlate of attitudes, awareness campaigns that focus only on changing what students think others believe may be insufficient. Interventions should additionally target the internalization of stigma—for example, brief cognitive restructuring modules that challenge beliefs equating help-seeking with personal inadequacy, self-compassion components, and contact-based sessions in which students hear peers with lived experience describe seeking help.

Second, the gender finding has direct programmatic implications. Male students reported both less positive attitudes and higher perceived public stigma, indicating that generic outreach is unlikely to reach them effectively. Services could deliver male-targeted outreach in settings where male students already gather (sports teams, engineering and law faculties, dormitories), use male peer ambassadors and male clinicians as visible models, frame services in terms of performance, stress management and problem-solving rather than emotional disclosure, and offer lower-threshold entry points such as brief single-session consultations, online self-help, or anonymous digital screening.

Third, because students with personal or family experience of psychiatric problems held more positive attitudes, peer-led programs that increase familiarity—for example, structured peer-support schemes or student mental-health ambassador networks—may be an efficient way to extend this “familiarity advantage” to students without such experience. Given the collectivist context, campaigns that also address families, rather than students alone, may be particularly relevant.

Finally, because stigma accounted for only a small share of the variance in attitudes, stigma-reduction should be embedded within broader strategies that also improve mental-health literacy, service visibility and accessibility, and confidentiality assurances. Future research should test these components experimentally, measure behavioral help-seeking outcomes rather than attitudes alone, and use longitudinal designs capable of testing the mediational chain implied by the self-stigma model and the Theory of Planned Behavior.

### Limitations

Several limitations should be noted. First, the cross-sectional design precludes causal inference and cannot capture change over time; longitudinal and intervention studies are needed, and the mediating role of self-stigma implied by our findings should be tested formally in longitudinal designs. Second, the single-university convenience sample, drawn from one city, limits generalizability and restricts variability in some key variables; cohort- or period-specific influences during the data-collection window cannot be excluded. Third, all constructs were measured by concurrent self-report, so shared-method variance may have inflated some associations. Fourth, the internal consistency of the self-stigma scale was low in this sample (α = 0.62). Because measurement error attenuates observed associations, the coefficients involving self-stigma—and, consequently, the variance explained by the models—are likely to be conservative underestimates of the true relationships; replication with a more reliable or culturally re-validated self-stigma measure is therefore a priority. Relatedly, the models explained a modest share of the variance in attitudes, which is consistent with the multidetermined nature of help-seeking rather than with model misspecification: attitudes are shaped by variables that were beyond the scope of the present design, including mental-health literacy, current symptom severity and perceived need, previous experience with services, anticipated benefits and risks of disclosure, subjective norms and perceived behavioral control as specified by the Theory of Planned Behavior, treatment-related beliefs, and structural factors such as service availability, cost, waiting times, and confidentiality assurances. Future studies incorporating these variables, together with more reliable stigma measurement, would be expected to account for a substantially larger proportion of the variance. Finally, the sample was relatively culturally homogeneous, and constructs such as stigma may be shaped by collectivism and family interdependence in ways that limit cross-cultural transfer.

## 5. Conclusions

Among Turkish university students, self-stigma and social stigma were negatively associated with attitudes toward seeking psychological help and positively associated with each other. In hierarchical regression, stigma explained a small but significant increment in variance beyond demographic characteristics, and self-stigma emerged as the only independent stigma-related correlate of attitudes—a pattern consistent with the proposition that public stigma operates largely through its internalization. Women and students with personal or family experience of psychiatric problems held more positive attitudes, whereas men reported greater social stigma. These associations suggest that stigma-reduction efforts in universities—particularly contact-based and cognitive approaches that address self-stigma, and outreach tailored to male students and to a collectivist family context—may help narrow the gap between need and help-seeking. Because the explained variance was modest, such efforts should be combined with broader improvements in mental-health literacy and service accessibility. Multi-center, longitudinal, and intervention-based studies incorporating behavioral help-seeking outcomes are needed to build on these findings.

## Figures and Tables

**Table 1:** Sociodemographic characteristics of the participants (*N* = 457).

Variable	*n*	%
**Gender**		
Female	286	62.6
Male	171	37.4
**Faculty**		
Medicine	90	19.7
Dentistry	87	19.0
Education	64	14.0
Law	51	11.2
Engineering	50	10.9
Science & Literature	33	7.2
Health Sciences	30	6.6
Other	52	11.4
**Monthly income**		
Low	61	13.3
Middle	258	56.5
High	138	30.2
**Family history of psychiatric disorder**		
Yes	68	14.9
No	389	85.1
**Previous psychiatric diagnosis**		
Yes	46	10.1
No	411	89.9

Note. Age: M = 20.6 years (SD = 2.3, range 16–37). Mother’s education: M = 9.3 years (SD = 4.7). Father’s education: M = 11.3 years (SD = 4.5).

**Table 2:** Scale scores by group: mean (SD).

Group	*n*	ATSPPH-SF	Self-Stigma	Social Stigma
**Gender**				
Female	286	62.09 (9.92)	27.24 (5.96)	9.92 (3.93)
Male	171	57.92 (11.63)	27.54 (5.88)	10.84 (3.60)
**Family history**				
Yes	68	64.12 (11.55)	26.50 (6.89)	10.76 (4.00)
No	389	59.90 (10.52)	27.50 (5.73)	10.17 (3.80)
**Previous psychiatric diagnosis**				
Yes	46	63.54 (10.58)	27.02 (6.28)	10.15 (4.14)
No	411	60.19 (10.75)	27.39 (5.89)	10.27 (3.80)
**Faculty**				
Medicine	90	60.22 (11.63)	26.36 (6.84)	8.76 (3.05)
Dentistry	87	62.64 (9.49)	27.67 (6.04)	10.20 (3.51)
Education	64	61.64 (12.70)	26.92 (6.34)	10.42 (3.96)
Law	51	59.39 (8.61)	28.78 (3.66)	10.67 (4.56)
Engineering	50	57.30 (7.45)	28.92 (3.91)	11.60 (3.05)
Science & Literature	33	58.55 (11.12)	27.42 (6.03)	10.88 (4.64)
Health Sciences	30	58.57 (7.60)	26.77 (5.24)	10.87 (4.21)
Other	52	62.77 (13.51)	26.48 (6.86)	10.37 (3.97)
**Monthly income**				
Low	61	62.08 (11.16)	26.11 (7.12)	9.23 (3.59)
Middle	258	60.54 (10.84)	27.36 (5.78)	10.29 (3.73)
High	138	59.82 (10.46)	27.89 (5.56)	10.67 (4.07)

Note. *t*-tests: ATSPPH-SF higher in women, t = 3.92, *p* < 0.001; social stigma higher in men, t = −2.54, *p* = 0.011. ATSPPH-SF higher with a family history, t = −2.81, *p* = 0.006, and a previous diagnosis, t = −2.03, *p* = 0.047 (others n.s.). ANOVA: by faculty, ATSPPH-SF F(7, 449) = 1.96, *p* = 0.059 (n.s.), self-stigma *p* = 0.14 (n.s.), social stigma F = 3.30, *p* = 0.002 (lowest in Medicine; Medicine vs. Engineering, Tukey *p* < 0.01); by income, ATSPPH-SF *p* = 0.39 (n.s.), self-stigma *p* = 0.15 (n.s.), social stigma F = 3.01, *p* = 0.050.

**Table 3:** Hierarchical multiple linear regression predicting attitudes toward seeking psychological help (ATSPPH-SF; *n* = 455).

Predictor	B	SE	β	t	*p*	95% CI
**Step 1: Demographic variables—R^2^ = 0.097, adj. R^2^ = 0.068, F(14, 440) = 3.38, *p* < 0.001**						
Age	0.272	0.217	0.058	1.25	0.210	[−0.15, 0.70]
Female (vs. male)	4.032	1.056	0.182	3.82	<0.001	[1.96, 6.11]
Mother’s education	−0.195	0.136	−0.086	−1.43	0.155	[−0.46, 0.07]
Father’s education	0.051	0.145	0.021	0.35	0.723	[−0.23, 0.34]
Income (Low → High)	−0.611	0.799	−0.036	−0.76	0.445	[−2.18, 0.96]
Family history (yes)	2.807	1.533	0.094	1.83	0.068	[−0.21, 5.82]
Prior diagnosis (yes)	2.618	1.851	0.074	1.41	0.158	[−1.02, 6.26]
Faculty: Dentistry	1.926	1.607	0.071	1.20	0.231	[−1.23, 5.08]
Faculty: Education	0.533	1.738	0.017	0.31	0.759	[−2.88, 3.95]
Faculty: Engineering	−1.446	1.879	−0.042	−0.77	0.442	[−5.14, 2.25]
Faculty: Health Sciences	−2.636	2.248	−0.061	−1.17	0.242	[−7.05, 1.78]
Faculty: Law	−2.034	1.876	−0.060	−1.08	0.279	[−5.72, 1.65]
Faculty: Science & Literature	−3.592	2.175	−0.087	−1.65	0.099	[−7.87, 0.68]
Faculty: Other	2.220	1.837	0.066	1.21	0.228	[−1.39, 5.83]
**Step 2: +Stigma variables—ΔR^2^ = 0.039, F-change(2, 438) = 9.91, *p* < 0.001; full model R^2^ = 0.136, adj. R^2^ = 0.105, F(16, 438) = 4.31, *p* < 0.001**						
Age	0.199	0.215	0.043	0.92	0.356	[−0.22, 0.62]
Female (vs. male)	3.835	1.043	0.174	3.68	<0.001	[1.79, 5.88]
Mother’s education	−0.253	0.135	−0.112	−1.88	0.061	[−0.52, 0.01]
Father’s education	0.131	0.143	0.054	0.91	0.361	[−0.15, 0.41]
Income (Low → High)	−0.344	0.786	−0.021	−0.44	0.662	[−1.89, 1.20]
Family history (yes)	2.516	1.513	0.084	1.66	0.097	[−0.46, 5.49]
Prior diagnosis (yes)	2.552	1.817	0.072	1.40	0.161	[−1.02, 6.12]
Faculty: Dentistry	2.446	1.583	0.090	1.55	0.123	[−0.67, 5.56]
Faculty: Education	1.034	1.720	0.034	0.60	0.548	[−2.35, 4.41]
Faculty: Engineering	−0.365	1.868	−0.011	−0.20	0.845	[−4.04, 3.31]
Faculty: Health Sciences	−2.222	2.219	−0.052	−1.00	0.317	[−6.58, 2.14]
Faculty: Law	−0.965	1.860	−0.028	−0.52	0.604	[−4.62, 2.69]
Faculty: Science & Literature	−2.801	2.157	−0.068	−1.30	0.195	[−7.04, 1.44]
Faculty: Other	2.539	1.814	0.076	1.40	0.162	[−1.03, 6.10]
Self-stigma	−0.313	0.089	−0.172	−3.50	<0.001	[−0.49, −0.14]
Social stigma	−0.178	0.140	−0.064	−1.27	0.205	[−0.45, 0.10]

Note. B = unstandardized regression coefficient; SE = standard error; β = standardized regression coefficient; t = t statistic; *p* = exact *p*-value for the corresponding coefficient; 95% CI = 95% confidence interval for the unstandardized coefficient B. Female and “yes” categories coded 1. Faculty was dummy-coded with Medicine as the reference category. ΔR^2^ denotes the increment in explained variance attributable to the two stigma variables entered in Step 2.

## Data Availability

The data presented in this study are available on request from the corresponding author. The data are not publicly available due to privacy and ethical restrictions, as participants did not consent to public sharing of their individual-level responses, in accordance with the approval of the Non-Interventional Clinical Research Ethics Committee of Gaziantep University.
